# Looking Beyond What You See: A Rare Cause of Dysphagia

**DOI:** 10.7759/cureus.38477

**Published:** 2023-05-03

**Authors:** Arshad M Muhammad, Mohammed F Farooqi, Mohamed M Bashir, Aysha S Aldhaheri, Adnan Agha

**Affiliations:** 1 Internal Medicine, Tawam Hospital, Al-Ain, ARE; 2 Department of Internal Medicine, College of Medicine and Health Sciences, United Arab Emirates University, Al-Ain, ARE; 3 Diabetes & Endocrinology, Tawam Hospital, Al-Ain, ARE

**Keywords:** overt hypothyroidism, thyroxine (t4), hypothyroidism, dysphagia, hashimoto's hypothyroidism

## Abstract

Hypothyroidism is a common endocrine condition with typical symptoms such as cold intolerance, weight gain, fatigue, constipation, and coarse skin, as well as less common symptoms such as depression, difficulty in concentration, and hair thinning. It is usually diagnosed by combining clinical features and applying clinical judgment; however, the wide spectrum of presenting symptoms can sometimes lead to a diagnostic dilemma. Dysphagia secondary to hypothyroidism is a rarely reported symptom in the literature and is believed to be associated with a hormonal effect on esophageal and gastric motility with neuromuscular incoordination; however, the underlying mechanism remains unknown. The most common cause of hypothyroidism is Hashimoto’s disease, which can rarely manifest as heartburn, possibly due to esophageal dysmotility. Herein, we describe an unusual presentation of severe hypothyroidism with dysphagia, for which we could not identify any obstructive cause despite extensive investigations This condition was resolved successfully with levothyroxine treatment. Through this report, we aimed to communicate to the audience the important learning message that hypothyroidism may cause symptoms of dysphagia and to inform practitioners regarding this possibility, which should be considered after ruling out any obstructive pathology.

## Introduction

Hypothyroidism is one of the most common endocrine conditions, with an estimated prevalence of around 3% in western countries [1]. Primary hypothyroidism caused by chronic autoimmune thyroiditis or Hashimoto’s disease is the most common cause of hypothyroidism and is typically characterized by the presence of thyroid peroxidase antibodies. A study in the United States revealed that these antibodies are present in 11% of the general population [2]. The clinical features of hypothyroidism include common symptoms such as cold intolerance, weight gain, fatigue, constipation, hair loss, and coarse skin as well as uncommon symptoms such as depression, difficulty in concentration, decreased libido, irregular menstrual cycles, and reduced fertility. Additionally, the clinical signs include bradycardia, cool extremities, features of pericardial effusions and entrapment syndromes (e.g., carpal tunnel syndrome), slowed relaxation of tendon reflexes, and rare neurological features, such as reversible cerebellar ataxia, dementia, psychosis, and myxedema coma [3]. A recent study from the Middle East showed that these well-known symptoms of hypothyroidism may not be prevalent in middle-aged patients, as some of them reported rare symptoms like dysphagia and dysarthria [4].

Dysphagia in hypothyroidism is usually reported with structural thyroid disease, which can cause dysphagia through pressure effect from the underlying enlarged goiter or secondary to lingual thyroid [5,6]. Lingual thyroid is an anatomical abnormality where the fetal thyroid gland fails to migrate in-utero and ends up being at the base of the tongue. However, this gland can enlarge at puberty or menopause due to an increase in thyroid hormone levels and may result in compressive symptoms [7]. Some cases reported in the literature, albeit rare, describe dysphagia in patients with severe hypothyroidism and older patients with a newly altered mental status, which is unrelated to structural thyroid disease. These symptoms can be improved via thyroid hormone replacement therapy [8]. Herein, we attempt to describe a rare presentation of hypothyroidism in the form of severe dysphagia, which was resolved after treatment with levothyroxine.

## Case presentation

A 21-year-old woman with no known history of previous medical ailments was presented to the emergency department with symptoms such as fatigue, hair loss, cold intolerance, poor concentration, weight gain, and excessive sleepiness, which started two months ago. Her other significant symptom was dysphagia to solid food and poor oral intake for two months. She was recently diagnosed with iron deficiency anemia and received intravenous iron one month before presentation; however, her fatigue symptoms did not improve. On physical examination, she was found to be mildly bradycardic with a heart rate of 51 beats per min and afebrile with a blood pressure of 103/59 mmHg and respiratory rate of 17 per min. She had mild eye puffiness, but no signs of anemia, jaundice, or dehydration were noted. Neck examination revealed no palpable goiter or any mass or thyroid bruit. Her cardiopulmonary examination was otherwise unremarkable. Neurological examination of the extremities indicated normal tone, power, and deep tendon reflexes of all extremities. Her clinical tests to induce fatigability showed negative results, and she did not have any cranial nerve palsy or ophthalmoplegia. Her bedside testing for dysphagia revealed overt signs of aspiration of sips of water. A speech and language pathologist evaluated the patient and indicated aspiration; therefore, she was recommended enteral feeds. The otolaryngology review indicated normal vocal cord movement.

Her laboratory findings showed severe hypothyroidism, with thyroid function tests revealing thyroid-stimulating hormone (TSH) level of 313 milli IU/L, T4 level of 0.6 pmol/L, and undetectable T3 along with the presence of strongly positive thyroid peroxidase antibodies. Other blood tests for extended electrolyte panel, celiac serology, iron profile, folate level, and vitamin B12 were all within normal limits (Table 1).

**Table 1:** Laboratory investigations on admission

Laboratory test	Value	Normal range
Sodium	139 mmol/L	136–146 mmol/L
Potassium	3.8 mmol	3.6–5.1 mmol/L
Chloride	107.3 mmol/L	98–107 mmol/L
Bicarbonate	19.5 mmol/L	22–29 mmol/L
Creatinine	109 micromole/L	45–84 micromole/L
Urea	2.4 mmol/L	2.67–8.07 mmol/L
Albumin	39 g/L	>21 g/L
Calcium	2.15 mmol/L	2.23–2.58 mmol/L
Amylase	76 units/L	28–100 units/mL
Lipase	35 IU/L	13–60 IU/L
Aspartate aminotransferase (AST)	20 IU/L	<32 IU/L
Alanine aminotransferase (ALT)	12 IU/L	<33 IU/L
Uric acid	173 micromole/L	142.8–339.2 micromole/L
Lactate dehydrogenase (LDH)	214 IU/L	135–214 IU/L
Transferrin	1.44 g/L	2.00–3.60 g/L
Transferrin saturation	38%	7%–42%
Total iron binding capacity (TIBC)	34 micromole/L	45–72 micromole/L
Thyroid-stimulating hormone (TSH)	303.700 milli IU/L	0.27–4.2 milli IU/L
Free T3	0.60 pmol/L	3.1–6.8 pmol/L
Free T4	0.5 pmol/L	12–22 pmol/L
Cortisol level	250 nmol/L	>200 nmol/L
Serum adrenocorticotropin (ACTH)	4.1 pmol/L	1.6–13.9 pmol/L
Haemoglobin	108 g/L	117–155 g/L
Mean carpuscular volume (MCV)	79.8 fL	81–100 fL
Thyroid peroxidase antibody (TPO Ab)	77.7 IU/mL	>34 IU/mL
Acetyl choline receptor (muscle) binding antibody	0.00 nmol/L	≤0.02 nmol/L

**Figure 1:**
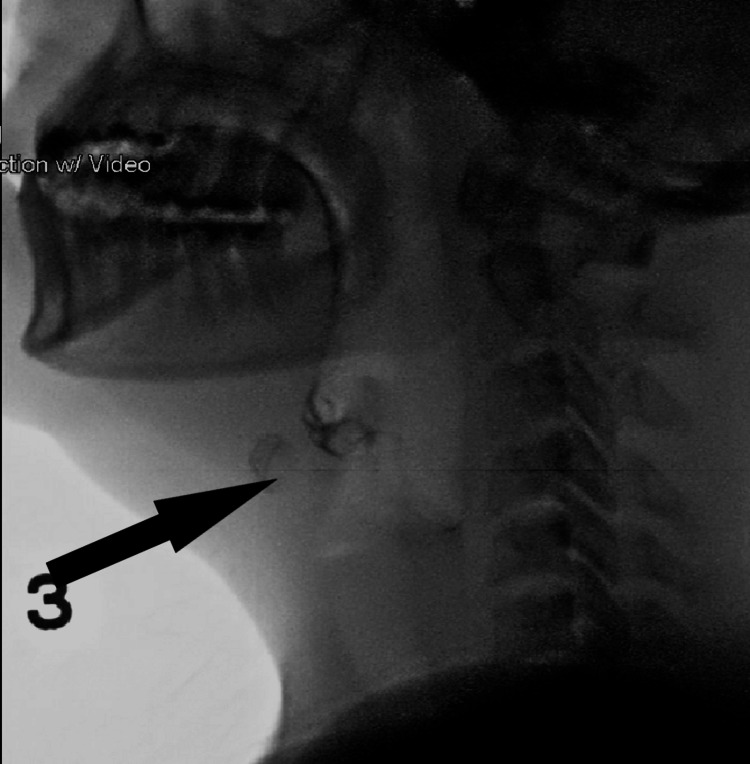
X-ray video fluoroscopy image with an arrow pointing toward the pooling of contrast in the valleculae

The patient was thoroughly evaluated for dysphagia via various imaging examinations, including swallow X-ray video fluoroscopy, which demonstrated pooling of contrast in the valleculae with evidence of penetration and trace aspiration (Figure 1).

Other imaging examinations, including thyroid gland ultrasound as well as neck and mediastinum computed tomography (CT), revealed no structural abnormalities and normal sized thyroid gland. Brain and cervical spine magnetic resonance imaging (MRI) was also performed to rule out any central causes of dysphagia, which excluded cranial nerve compression or other lesions that could cause bulbar symptoms. Her upper gastrointestinal endoscopy was normal, and gastric biopsies revealed mild-to-moderate chronic active gastritis with *Helicobacter pylori* infection, with no signs of metaplasia, dysplasia, or malignancy. Electromyography and nerve conduction studies revealed that her dysphagia was not due to a central neurological cause like motor neuron disease, and she only had mild right carpel tunnel syndrome. Myasthenia gravis was ruled out by an expert neurologist based on the absence of acetylcholine receptor antibody.

She was initially treated with intravenous levothyroxine (100 mcg) until a nasogastric (NG) tube was used for feeding. The levothyroxine dose was maintained at 150 mcg daily via the NG route, which significantly improved her TSH level to 173 milli IU/L within one week of treatment. After two weeks of treatment, the speech and language pathologist was reconsulted for a swallowing assessment, which revealed that her dysphagia had improved and there were no signs of aspiration, as confirmed via. Further, the patient was allowed to consume an oral diet with pureed consistency, and the NG tube was removed. She was discharged and treated with oral levothyroxine; subsequently, at a three-week follow-up, she showed complete resolution of all dysphagia symptoms. She remained asymptomatic and her thyroid function results were within normal limits at a three-month outpatient follow-up (Table 2).

**Table 2:** Trend of thyroid function results in response to levothyroxine treatment from day 0 (first presentation) to day 90 (at outpatient follow-up) * NA = Not available (T4 on day 7 was not processed)

Lab results	Day 0	Day 7	Day 30	Day 90
Thyroid-stimulating hormone (TSH) milli IU/L	303.7	173	0.378	2.120
Free T4 pmol/L	0.6	NA*	34.3	18.2

## Discussion

This case describes a rare cause of dysphagia secondary to severe hypothyroidism. Our patient presented with symptoms of hypothyroidism, and biochemical testing revealed significantly elevated TSH levels of >300 milli IU/L (Table 1). As the patient began thyroid hormone replacement therapy via intravenous and then NG routes, her TSH levels decreased, which corresponded with her clinical symptoms of dysphagia. The patient was discharged and treated with oral levothyroxine; subsequently, at a four-week follow-up, her TSH levels returned to near-normal limits and dysphagia was completely resolved. The patterns of TSH decline and T4 increase are presented in Table 2. At a three-month follow-up, her TSH and T4 levels remained within normal limits, and no further dysphagia or swallowing problems were noted. Therefore, the dysphagia was most likely caused by severe hypothyroidism, which was reversed after thyroxine replacement therapy. Notably, differentials such as neuromuscular disorders, bulbar and pseudobulbar palsy, and acetylcholine receptor antibody tests were ruled out by an expert neurologist team based on extensive testing, which included brain MRI, cervical spine MRI, electromyography, nerve conduction studies, and acetylcholine receptor antibody tests. The differential for structural causes of dysphagia was ruled out by comprehensive imaging and testing, which included esophagogastroduodenal endoscopy, neck and thyroid ultrasound, and neck and upper thorax CT. Improvement in dysphagia symptoms with levothyroxine treatment was reassuring, and this phenomenon has previously been discussed in the literature in cases of severe hypothyroidism and myxedema [9].

Neuromuscular manifestations in the context of autoimmune hypothyroidism can be observed with more frequently reported abnormalities, such as mild-to-moderate myopathy and sensorimotor neuropathy, with carpal tunnel syndrome being the most prevalent neuropathy. This syndrome was also detected in our patient via a nerve conduction test [10]. However, she was asymptomatic and showed no signs on clinical examination. Neuromuscular manifestations are usually reversed after appropriate restoration of thyroid function [10,11], which was also observed in our case.

A previous study suggested that gut motility disorders are the first and only presenting signs of thyroid gland disorders, which may be due to the interaction between thyroid hormones, particularly between thyronine and catecholamines on muscle cell receptors in the gastrointestinal tract [12]. Hypothyroidism is believed to cause an alteration of the anatomical structure of the esophagus or alteration of neuromuscular functions and peristalsis, resulting in delayed gastric emptying and reduced acid production, a feature commonly associated with autoimmune disease, which usually resolves after normalization of thyroid function [13].

## Conclusions

Hypothyroidism is a common medical condition, and clinicians should consider that dysphagia may be an uncommon presentation of hypothyroidism. The most common etiology of dysphagia in thyroid disorders is obstruction with external compression due to goiter; however, in our case, obstruction was completely ruled out via clinical examination, imaging, and endoscopy. Therefore, we believe that dysphagia was secondary to neuromuscular incoordination due to severe hypothyroidism.

Our case also demonstrates that dysphagia caused by hypothyroidism responds well to levothyroxine treatment, as our patient recovered completely after receiving levothyroxine replacement therapy.
